# What is the effect of sensory discrimination training on chronic low back pain? A systematic review

**DOI:** 10.1186/s12891-016-0997-8

**Published:** 2016-04-02

**Authors:** Samuel Kälin, Anne-Kathrin Rausch-Osthoff, Christoph Michael Bauer

**Affiliations:** Institute of Physiotherapy, Department of Health, Zurich University of Applied Sciences, Technikumstrasse 71, 8400 Winterthur, Switzerland; University of Tampere, School of Medicine, Kalevantie 4, FI-33014 Tampere, Finland

**Keywords:** Low Back pain, Sensory feedback training, Physical therapy, Systematic review, Rehabilitation

## Abstract

**Background:**

Sensory discrimination training (SDT) for people with chronic low back pain (CLBP) is a novel approach based on theories of the cortical reorganization of the neural system. SDT aims to reverse cortical reorganization, which is observed in chronic pain patients. SDT is still a developing therapeutic approach and its effects have not been systematically reviewed. The aim of this systematic review was to evaluate if SDT decreases pain and improves function in people with CLBP.

**Methods:**

A systematic review was performed on the available literature to evaluate the effects of SDT. Randomised controlled trials compared the effectiveness of SDT on pain and function in people with CLBP with the effectiveness of other physiotherapy interventions, no treatment, or sham therapy. The methodological quality of the included studies and the clinical relevance of reported treatment effects were investigated.

**Results:**

The original search revealed 42 records of which 6 fulfilled the inclusion criteria. The majority of studies showed that SDT caused statistically significant improvements in pain and function, but only two studies reported clinically relevant improvements. The applied SDT varied considerably with regard to dosage and content. The methodological quality of the included studies also varied, which hampered the comparability of results.

**Conclusions:**

Although SDT seems to improve pain and function in people with CLBP, study limitations render firm conclusions unsafe. Future studies should pay closer attention to power and sample selection as well as to the content and dosage of the SDT intervention. We recommend a large, well-powered, prospective randomized control study that uses a standardized SDT approach to address the hypothesis that SDT causes clinically relevant improvements in pain and function.

**Electronic supplementary material:**

The online version of this article (doi:10.1186/s12891-016-0997-8) contains supplementary material, which is available to authorized users.

## Background

Chronic low back pain (CLBP) has been associated with neurochemical, structural, and functional cortical changes [1] of several brain regions including the somatosensory cortex. Those changes have been observed in people with CLBP [1], phantom limb pain [2] and chronic regional pain syndrome (CRPS) [3–5] and manifest in medialization and expansion of the cortical representation of the low back in the primary and secondary somatosensory cortex [1, 6] and are commonly described as “cortical reorganization” [7]. Cortical reorganization is paralleled by increased pain levels and decreased tactile acuity [8], a clinical symptom also found in people with arthritis, CRPS, and CLBP [9].

Cortical reorganization presents a barrier to successful recovery; however the plasticity that underpins cortical reorganization also suggests that it might be responsive to targeted treatments [10], such as sensory discrimination training (SDT). SDT comprises tactile discrimination [11] and sensorimotor retraining [12] approaches, which involve the recognition of the location and the type of the stimuli by the patient (localization training). These treatment approaches improve tactile acuity, normalize cortical reorganization and decrease pain in patients with CRPS, chronic limb pain, and phantom limb pain [2–4, 6, 11]. They are based on localization training and apply a combination of various sensory stimuli to different locations [11]. Instead of comprising a passive and repetitive stimulation of the affected area or of another body part, such treatments require active perception and localization of the stimulus (discrimination component) by the patient. However, these approaches are not fully developed from a pathoanatomical perspective [1], since the processes involved in cortical reorganization in CLBP are not fully understood [13].

A previous systematic review [14] has considered studies involving various sensory feedback training approaches, such as SDT [15, 16], visualisation of the painful area [17], or motor control exercises [18] and their effect on pain intensity and disability in people with CLBP. It concluded that, while preliminary results are encouraging, further systematic evidence on SDT is needed to gain knowledge of its long-term effectiveness on CLBP and to optimise treatment protocols [14].

Furthermore, while there is a growing body of research on the effectiveness of various types of SDT stimuli that are delivered to people with CLBP, ranging from acupuncture [19] to vibration [15], currently there is no systematic evidence on the effectiveness of SDT compared to other approaches, nor on the superiority of one SDT type above the others. Furthermore, SDT has been compared to a wide spectrum of alternative therapies, ranging from no treatment [20] to electrostimulation without a discrimination component [15]. A systematic review of the literature has not yet been previously conducted. This systematic review, based on a literature search for further evidence, aimed to determine the effectiveness of SDT, in terms of clinically relevant measures such as pain intensity and function.

## Methods

This systematic review followed guidance from the Centre for Reviews and Disseminations’ [21] and the *Cochrane Handbook for Systematic Reviews of Interventions* [22] for undertaking reviews in health care. A completed PRISMA checklist is provided in Additional file 1.

### Data sources and searches

Only randomized controlled trials (RCT) were included in this systematic review. Study identification commenced with an electronic search, using the MEDLINE (through PubMed), CINAHL, EMBASE, and Cochrane Libraries, to identify articles published until August 2015, in English or German (see Additional file 2 for search example). Search terms used were *randomized controlled trial*, *chronic pain, back*, *low back*, *lower back*, *lumbar spine*, *lumbar column*, *sensory feedback*, *sensory training*, *sensorimotor, sensory motor training*, *sensory motor feedback*, *feedback training*, sensory *discrimination training*, *sensorimotor training*, *sensorimotor retraining*, *tactile stimulation*, *perceptive rehabilitation*, and *tactile discrimination*. A combination of these terms was used to extract a comprehensive list of articles, from which the titles and abstracts were screened for eligibility. An additional search was conducted for grey literature on issue-specific databases, [23–25] based on citation tracking and key author searches.

### Eligibility criteria

The following criteria determined the eligibility of each study for inclusion in the systematic review: RCT, published in English or German. The participants had to be 18 years or older and match with the following inclusion criteria: CLBP of at least 3 months’ duration [26], no red flag disorders or specific pathology [27, 28], no coexisting major medical disease, and no spinal surgery in the last 12 months.

Studies were included if they used SDT that was either applied manually, with machines, or with other tools used to employ sensory inputs. The main content (i.e. more than 50 %) of the therapy program studied must be SDT, consisting of the active perception of the stimulated body part. SDT must be compared with exercise, placebos, sham therapy, no therapy, passive treatment (such as ultrasound or electrotherapy), or SDT combined with other therapies. Studies were included if they assessed, on a symptoms level [29], self-reported pain intensity with a validated method such as the visual analogue scale, the numerical rating scale, or the pain rating index. Furthermore, studies were included that used a validated method to measure measuring physical functionality, on the level of daily functioning [29]. Studies were included that reported either or both self-reported pain intensity and daily functioning. Two reviewers independently evaluated records for eligibility. Disagreement was resolved by discussion and consensus. Reported arbitration would have been applied by another person if it had been required. To avoid duplication in pooling, data were included only once if they were reported in previously published work.

### Quality assessment

Two reviewers independently analysed the quality of the included studies using the Physiotherapy Evidence Database (PEDro) [30] tool to assess the risk of bias [31, 32]. Discrepancies were solved by consensus.

Clinical relevance was assessed using the Cochrane Collaboration Back Review Group’s method guidelines for systematic reviews [33], which consist of the following five questions:

1. Are the patients described in detail so that you can decide whether they are comparable to those whom you see in your practice?
2. Are the interventions and treatment settings described well enough so that you can provide the same for your patients?
3. Were all clinically relevant outcomes measured and reported?
4. Is the size of the effect clinically important?
5. Are the likely treatment benefits worth the potential harms?

### Data analysis

**Fig. 1:**
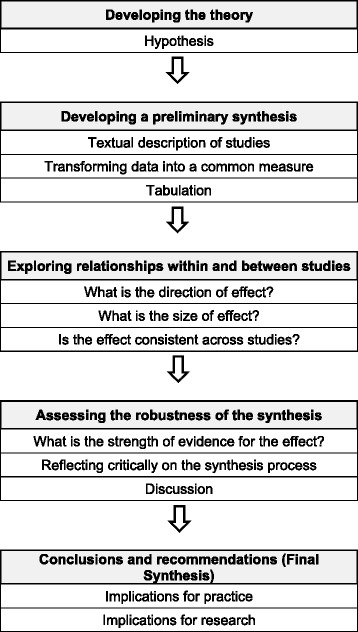
Framework of the systematic review

Two reviewers independently extracted information from each study, including the setting of the study, characteristics of patients, inclusion and exclusion criteria, including the use of SDT instrumentation, intervention and control intervention, study protocol, and outcomes (pain and function). The primary analyses, which were defined a priori were included: SDT compared to no treatment or to sham therapy, SDT compared to another intervention, and SDT added to an intervention compared to the intervention without SDT. Due to the expected methodological diversity of the studies it was decided a priori to analyse the findings using a qualitative narrative synthesis approach instead of a quantitative synthesis approach, according to the recommendations by the Cochrane group [26]. The qualitative narrative synthesis of this systematic review was structured according to the Centre for Reviews and Disseminations’ guidance for undertaking reviews in health care [21] and the *Cochrane Handbook for Systematic Reviews of Interventions* [22]. Figure 1 shows the detailed framework chosen for the narrative synthesis. Different outcome measures to rate pain and function were rescaled from 0 to 100 units for each outcome measure [34]. Improvements of 20 units out of 100 in pain [35, 36] and 10 out of 100 in function [32, 36] were considered the minimal clinically important differences (MCID).

## Results

**Fig. 2:**
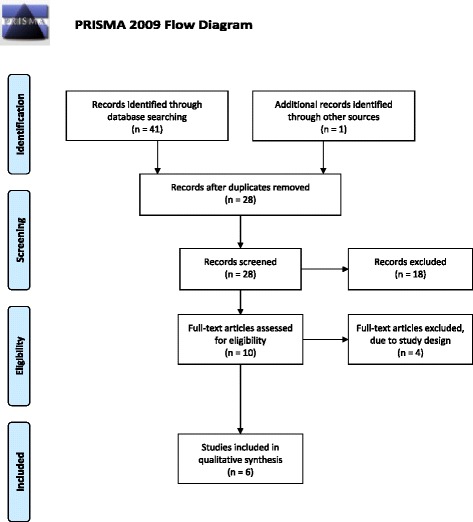
Flow diagram

The search revealed 42 records; 28 of which were screened in abstracts after duplicates were removed, and 10 of which were screened in full text (Fig. 2). The remaining four studies were excluded due to study design. Six studies [15, 16, 20, 37–39] with 257 patients fulfilled the inclusion criteria. Both the intervention and control groups of one study [39] received SDT and were therefore handled as a comparison of two different intervention groups.

Additional file 3 summarizes the applied methods, participant’s characteristics, interventions, and outcome measures of the included studies. Table 1 shows the methodological quality assessment for risk of bias. The methodological quality assessment revealed that all studies except one [37] were of moderate or high quality (≥5/10 points on the PEDro scale) [30]. Table 2 summarizes the assessment of clinical relevance. The criteria for inclusion and exclusion, the content of the interventions, and the clinical settings have been poorly documented in two studies, threatening external validity and applicability to clinical practice [15, 20]. Pain and dysfunction have been inadequately reported in one study, for the post-treatment assessments [15], or were not reported at all for dysfunction [37]. No serious adverse effects have occurred in two studies, indicating lack of adverse events [20, 38]. In the remaining studies adverse effects have not been discussed, indicating lack of evidence regarding adverse effects [15, 16, 37, 39]. The “Surface for perceptive rehabilitation” (Su-Per) treatment applied in three studies [16, 37, 39] requires special equipment, the costs of which are not reported. Table 3 provides the results of each study at each time point as well as the between-groups comparisons. Only two studies provided long-term results, one for 12 and the other for 24 weeks after treatment [16, 39].

**Table 1:** Methodological quality assessment according to PEDro [30]

PEDro criteria	Barker et al. (2008) [15]	Hohmann et al. (2012) [20]	Morone et al. (2011) [16]	Paolucci et al. (2012) [37]	Ryan et al. (2014) [38]	Vetrano et al. (2013) [39]
Eligibility criteria specified	Yes	Yes	Yes	Yes	Yes	Yes
Random allocation	Yes	Yes	Yes	Yes	Yes	Yes
Concealed allocation	Yes	Yes	Yes	No	Yes	Yes
Similar groups at baseline	Yes	Yes	Yes	No	Yes	Yes
Blinding of subjects	No	No	No	No	No	No
Blinding of therapists	No	No	No	No	No	No
Blinding of assessors	Yes	No	Yes	No	No	Yes
Measure of one key outcome obtained for 85 % of subjects	Yes	Yes	Yes	No	Yes	Yes
Intention-to-treat analysis	Yes	Yes	Yes	No	No	Yes
Between-group comparisons of at least one key outcome	Yes	Yes	Yes	Yes	Yes	Yes
Point and variability measures of at least one key outcome	Yes	Yes	Yes	Yes	Yes	Yes
Total Score	8	7	8	3	6	8

**Table 2:** Clinical relevance assessment according to Cochrane [33]

Studies	Patients	Inter- ventions	Relevant outcomes	Size of effect	Benefits and harms	Factors influencing the clinical relevance
Barker et al. (2008) [15]	YES	NO	NO	NO	YES	Patients: Pre-treatment surgery is poorly described. Intervention is poorly described. Results 6 weeks and 12 weeks post-treatment are not integrated.
Hohmann et al. (2012) [20]	NO	NO	YES	NO	YES	Patients: Pregnancy is poorly described, although 15 patients out of 21 were women. Intervention is poorly described.
Morone et al. (2011) [16]	YES	YES	YES	YES	YES	Patients: Pre-treatment surgery is poorly described.
Paolucci et al. (2012) [37]	YES	YES	NO	NO	YES	Patients: Pre-treatment surgery is poorly described. Function is no outcome measure.
Ryan et al. (2014) [38]	NO	YES	YES	NO	YES	Patients: Pre-treatment surgery or pregnancies are poorly described. Ratio men / women is not documented.
Vetrano et al. (2013) [39]	YES	YES	YES	YES	YES	There have been remarkably more women included in this study than men. Intervention 2 (Control group) received also a SFT.

*Abbreviations*: *SFT* sensory feedback training

**Table 3:** Outcomes

study	groups	pre		post		4 weeks post		12 weeks post		24 weeks post		change pre / post		between-group comparisons pre / post	
		pain	function	pain	function	pain	function	pain	function	pain	function	pain	function	Pain	Function
		Mean (SD) units	Mean (SD) units	Mean (SD) units	Mean (SD) units	Mean (SD) units	Mean (SD) units	Mean (SD) units	Mean (SD) units	Mean (SD) units	Mean (SD) units	Mean (CI) units	Mean (CI) units	Difference in units	Difference in units
Barker et al. (2008) [15]	Intervention group	63 (19)	40.8 (15.9)	55 (18)	40.2 (8.7)	-	-	-	-	-	-	- 8 (−15 to −1)	- 0.6 (−3.8 to 2.7)	1 (*p* = 0.83) *	- 0.3 (*p* = 0.85) *
	Control group	66 (14)	42.8 (14.8)	59 (14)	41.9 (5.1)							- 7 (−13 to −1)	- 0.9 (−3.0 to 1.1)		
Hohmann et al. (2012) [20]	Intervention group	50 (23)	22.8 (14.5)	32 (22)	18.8 (14.6)	-	-	-	-	-	-	- 18 (*+)	- 4.0 (*+)	**13 (** ***p*** **= <0.001)**	- 1.1 (*p* = 0.878)
	Control group	49 (19)	25.0 (13.9)	54 (19)	19.9 (10.9)							+ 5 (*+)	- 5.1 (*+)	CI: −23 (−32 to −13)	CI: 0.4 (−4.8 t 5.6)
Morone et al. (2011) [16]	Intervention group	60 (10)	34 (20)	40 (20)	16 (16)	-	-	50 (10)	16 (12)	50 (40)	20 (19)	- 20 (*+)	- 18 (*+)	**10 (** ***p*** <0.001) *	8 / 16
	Control group 1	70 (20)	26 (24)	60 (40)	16 (18)			50 (40)	12 (16)	40 (40)	10 (12)	- 10 (*+)	- 10 (*+)	(Intervention group compared with control group 1 and control group 2)	(*p* = 0.403) * (Intervention group compared with control group 1 and control group 2)
	Control group 2	70 (20)	24 (20)	80 (10)	22 (24)			80 (10)	26 (20)	70 (30)	26 (18)	+ 10 (*+)	- 2 (*+)		
Paolucci et al. (2012) [37]	Intervention group	40 (15)	-	23 (14)	-	-	-	-	-	-	-	- 17 (*+)		- 2 (*p* = 0.436) *	
	Control group	51 (32)		32 (13)								- 19 (*+)			
Ryan et al. (2014) [38]	Intervention group	49 (19)	38.8 (27.5)	40.9 (27.8)	31.7 (31.7)	-	-	-	-	-	-	- 8.1 (*+)	- 7.1 (*+)	- 24.8 (*p* = 0.056)	- 9.5 (*p* = 0.237)
	Control group	48 (31)	30.4 (12.9)	15.2 (14.5)	13.8 (14.1)							- 32.8 (*+)	- 16.6 (*+)	CI: 25.6 (−0.7 to 51.9)	CI: 2.2 (−1.6 to 6.0)
Vetrano et al. (2013) [39]	Intervention group 1	75 (21)	28 (14.5)	50 (35)	12 (11)	40 (35)	12 (9)	20 (55)	7 (13.5)	-	-	- 25 (*, ***p*** **= 0.002)**	- 16 (*, ***p*** **= 0.003)**	5 (*p* = 0.179) *	2 (*p* = 0.299) *
	Intervention group 2	50 (30)	24 (16)	30 (20)	10 (8)	30 (15)	10 (8)	20 (20)	4 (11)			- 20 (*, ***p*** **< 0.001)**	- 14 (*, ***p*** **< 0.003)**		

*Abbreviations*: *SD* Standard Deviation; *p p*-value, *CI* 95 % Confidence Interval, * = CI not reported, + = *p*-value not reported, in bold print = difference is greater than the minimal clinical important difference

Results of [18] have been calculated by the authors using raw data

Pain and function data have been transformed into common measure (see Methods)

In general, each SDT intervention led to the decrease in pain and the improvement of function [15, 16, 20, 37–39]. Five control interventions also resulted in decreased pain levels [15, 16, 37, 38], while two control interventions actually resulted in an increase of pain [16, 20]. All control interventions led to improvement of function [15, 16, 20, 37, 38].

Three STF interventions [16, 39] and one control intervention [38] resulted in a clinically relevant decrease of pain intensity. Furthermore, three SDT [16, 39] and two control interventions [16, 38] triggered a clinically relevant improvement in function (Table 3). The two waiting list control interventions showed an increase in pain and an improvement of function that is below the MCID [16, 20].

The improvement in pain after the intervention differed significantly between the groups in two studies [16, 20] that favoured SDT over control interventions, whereas no study observed significant differences between the groups for function. The two studies with a follow-up found no significant between-groups differences for changes in pain or function [16, 39] after 12 and 24 weeks, respectively.

One study used sham therapy as a control intervention, which has shown a decrease of 32.8 units in pain and 16.6 in function, which are the highest clinically relevant improvements of all included studies [38]. This sham therapy was superior to SDT (tactile acuity training), but there was no significant difference between the groups. The sham therapy closely resembled the SDT, the difference being that the participants were not asked to focus on the stimuli in the control group [38]. The sham therapy group and the SDT group (each *n* = 12) each had a small sample size of six, with three dropouts.

One study used transcutaneous electrical nerve stimulation (TENS) [15] and two studies used back school programs as control interventions [16, 37]. None of these interventions led to a clinically relevant improvement in pain, but one of the back school programs led to the improvement of function [16].

## Discussion

The aim of this systematic review was to determine the effect of SDT on pain and function in people with CLBP. The collected data of the six included studies suggest that there is no conclusive evidence about the effectiveness of SDT in people with CLBP. All SDT approaches reported a reduction in pain and function [15, 16, 20, 37–39], but this was inconsistent, as some approaches were either not significantly superior in comparison to the control group [15, 37] or even inferior [38] in the short term. It is impossible to draw conclusions about the long-term effects of SDT since only two studies [16, 39], reported on those effects and found no significant between-groups differences.

SDT is a very broad term covering a range of different therapies, which are highlighted in this systematic review. The duration of the sessions and treatments varied widely and were not always reported in detail [15, 20]. Great heterogeneity concerning the types of intervention has been observed, which ranged from self-dependence [15, 20], to the help of a formal or informal caregiver [38], and to a full applied treatment by a health professional [16, 37, 39]. Considerable differences in physical activity levels during the treatments were observed, ranging from no integration of physical activity [20] to SDT combined with physical activities [16, 37, 39]. The studies used a different starting position and applied dissimilar devices [15, 16, 20, 37–39].

One recent crossover design study compared acupuncture with optimized sensory discrimination to standard acupuncture [19]. A greater decrease in pain with significant between-groups differences was observed when acupuncture with optimized sensory discrimination was applied, indicating that acupuncture may offer an additional benefit if combined with SDT. Another excluded study with a single-case design with three participants used a sensorimotor retraining approach consisting of a graded sensory and motor retraining [12]. A decrease in pain and increase in function was reported, supporting the findings of the included studies [15, 16, 20, 37–39].

### Utility

The Su-Per treatment, which is a form of SDT applied in four intervention groups [16, 37, 39], requires special equipment, the price of which is not reported but nevertheless implies investment for the therapist and hence reduced applicability for home use. This increases the dependency of the patient on the therapist or an assistant and does not allow for the treatment to be integrated easily into daily life, and therefore it hampers practicability. Treatment that is applied by a therapist or other caregiver might evoke or enlarge the illness behaviour by offering excessive help [26], meaning that a patient develops the tendency to make use of unnecessary assistance. In this light, a self-administered intervention might be an interesting alternative. If patients could conduct their treatment following individually tailored schedules it might increase their adherence to treatment protocols. One SDT intervention was administered at home by an informal caregiver [38]. This might increase the practicality, by decreasing the patients’ dependency on the therapists. However, it might also reduce the ability to verify and quantify the results of the applied therapy, especially if no guidance is given about the frequency of the application and if the received therapy dosage is inaccurately reported [15, 20]. Furthermore, no clinically relevant improvements in pain or function were observed in these studies [15, 20, 38]. Alternative interventions, such as acupuncture, showed improvements in pain and function and have so far been studied in a crossover design study [19]. However these approaches require a fully trained health professional. Two SDT interventions [15, 20] and the TENS control intervention [15] have been applied at the patients’ home by themselves. SDT design should be easily applicable to daily life and it should limit expenses for therapists and patients alike.

### Study limitations

The limitations of the included studies make firm conclusions unfeasible. The risks of bias have been detected in the PEDro analysis, namely two categories: Blinding of: Blinding of (1) the subjects and (2) the therapists has not been realized in any of the included studies [15, 16, 20, 37–39]. Other measures to control for the risk of bias are poorly described, such as random allocation, which in one study resulted in group differences for a more disabled intervention group (eight points on the standardized scale) [16]. Another study used sham therapy as control intervention, which closely resembled the SDT that was received in the intervention group [38]. A control group with greater contrast to the intervention group could include physical activity or no treatment and control for expectation bias. In addition, the included studies recruited rather small samples, ranging from 32 to 75 patients. One study acknowledged that the authors would benefit financially if the FairMed® device would reach the market [15]. The studies applying the Su-Per treatment or needle stimulation did not declare whether competing interests existed [16, 20, 37, 39]. While the included studies investigated the effects of tactile acuity training [15, 16, 20, 37–39], they did not examine its effect on cortical representation of the lower back, and only one study conducted sensory testing [20]. This is a serious limitation and undermines their overall findings as it prevents any conclusions being drawn concerning the neurophysiological mechanisms underlying any treatment effect.

### Recommendations for future research

Well-powered studies with sufficiently large sample sizes are necessary to verify the observed effect of SDT on pain and function and to compare different forms of SDT. Measures of pain and function should be accompanied by measures of tactile acuity and cortical representation to explain the neurophysiological mechanisms underlying any treatment effects in the short and long term. Furthermore, future studies should explore which SDT components might complement other treatment approaches after individual persons with CLBP are screened for the dominant factors driving their pain state [27, 28] . Such studies should also determine if certain participants, for example those with predominant sensory impairment in the painful area, might be more likely to benefit from SDT. Patients suffering from kinesiophobia and sensory impairment in the painful area might benefit from combined visualization of lumbar movements [17] motor control exercises [18] and SDT [15, 16, 20, 37–39]. It might also be of interest to explore whether SDT complemented by visual feedback, such as the visualization of the painful area, has a beneficial effect on treatment outcomes. In the majority of the studies, the patients lay on their back without receiving visual feedback [16, 20, 37, 39]. Barker et al. (2008) utilized a prone lying position, with a picture of the patients’ own back as an additional sensory input [15]. Experiments on healthy participants using visual feedback showed no improvement of tactile acuity of the lower back [40], but this approach could be re-evaluated by assessing people with CLBP with impaired tactile acuity. Patients’ and therapists’ acceptance of SDT devices and patients’ adherence to their use are other important aspects that should be addressed.

## Conclusions

While SDT appears to improve pain and function in people with CLBP, there is conflicting evidence from 6 trials (257 people) [15, 16, 20, 37–39] about whether SDT is more effective in the short term compared to another intervention, no treatment, or sham therapy. Larger, well-powered, prospective RCTs with long-term follow-ups are recommended. Such studies should pay close attention to the risks of bias and to applicability in clinical practice.

### What is already known on this topic

Cortical changes and sensory impairments, such as decreased tactile acuity observed in people with CLBP are neurochemical, structural, and functional [1], are paralleled by sensory impairments such as decreased tactile acuity, and are similar to those observed in phantom limb pain [2] and chronic regional pain syndrome patients [10]. Cortical reorganization in chronic pain states can generally be treated with sensory feedback training [2, 4, 6, 11]. There is a growing body of research on the effect of various types of sensory feedback training, including SDT, people with CLBP [15, 16, 19, 20, 37–39].

### What this study adds

Currently there is no conclusive evidence on the effectiveness of SDT on CLBP compared to other approaches. This SR contributes to a better understanding of the effectiveness of SDT, in terms of clinically relevant measures such as pain intensity and function. SDT improves pain and function in people with CLBP but there is conflicting evidence whether it is more effective than other interventions.

### Ethics approval and consent to participate

Not applicable.

### Consent for publication

Not applicable.

### Availability of data and materials

All data is contained within the manuscript or the additional files.

## Additional files

Additional file 1: PRISMA 2009 Checklist. (DOC 62 kb) Additional file 2: Pubmed search string example. (DOCX 17 kb) Additional file 3: Summary of included studies. (DOCX 28 kb)

## Abbreviations

CLBP: chronic low back pain
CRPS: chronic regional pain syndrome
MCID: minimal clinically important differences
PEDro: physiotherapy evidence database
RCT: randomized controlled trial
SDT: sensory discrimination training
Su-Per: surface for perceptive rehabilitation
TENS: transcutaneous electrical nerve stimulation
