# Supplementary material for: What is the effect of sensory discrimination training on chronic low back pain? A systematic review
Source: BMC Musculoskelet Disord. 2016 Apr 2;17:143. doi: 10.1186/s12891-016-0997-8 (PMC4818915; doi:10.1186/s12891-016-0997-8)
Supplement: Additional file 2: — Pubmed search string example. (DOCX 17 kb) [file 12891_2016_997_MOESM2_ESM.docx]

# Pubmed search string example

Search#, Query, Items found

#23,"Search (((#1) AND #2) AND #8) AND #22 Filters: Publication date from 1900/01/01 to 2015/08/31; Humans",20

#22,"Search ((((((((((((#9) OR #10) OR #11) OR #12) OR #13) OR #14) OR #15) OR #16) OR #17) OR #18) OR #19) OR #20) OR #21 Filters: Publication date from 1900/01/01 to 2015/08/31; Humans", 28292

#21,"Search sensorimotor retraining Filters: Publication date from 1900/01/01 to 2015/08/31; Humans", 24

#20,"Search sensorymotor retraining Filters: Publication date from 1900/01/01 to 2015/08/31; Humans", 0

#19,"Search sensorimotor training Filters: Publication date from 1900/01/01 to 2015/08/31; Humans", 885

#18,"Search sensorymotor training Filters: Publication date from 1900/01/01 to 2015/08/31; Humans", 2

#17,"Search tactile discrimination Filters: Publication date from 1900/01/01 to 2015/08/31; Humans", 1138

#16,"Search perceptive rehabilitation Filters: Publication date from 1900/01/01 to 2015/08/31; Humans", 96

#15,"Search tactile stimulation Filters: Publication date from 1900/01/01 to 2015/08/31; Humans", 2952

#14,"Search sensory discrimination training Filters: Publication date from 1900/01/01 to 2015/08/31; Humans", 368

#13,"Search feedback training Filters: Publication date from 1900/01/01 to 2015/08/31; Humans", 17232

#12,"Search sensory motor feedback Filters: Publication date from 1900/01/01 to 2015/08/31; Humans", 1808

#11,"Search sensory motor training Filters: Publication date from 1900/01/01 to 2015/08/31; Humans", 1400

#10,"Search sensory training Filters: Publication date from 1900/01/01 to 2015/08/31; Humans",4309

#9,"Search sensory feedback Filters: Publication date from 1900/01/01 to 2015/08/31; Humans", 3853

#8,"Search ((((#3) OR #4) OR #5) OR #6) OR #7 Filters: Publication date from 1900/01/01 to 2015/08/31; Humans", 156221

#7,"Search lumbar column Filters: Publication date from 1900/01/01 to 2015/08/31; Humans", 1883

#6,"Search lumbar spine Filters: Publication date from 1900/01/01 to 2015/08/31; Humans", 52814

#5,"Search lower back Filters: Publication date from 1900/01/01 to 2015/08/31; Humans", 12970

#4,"Search low back Filters: Publication date from 1900/01/01 to 2015/08/31; Humans", 30615

#3,"Search back Filters: Publication date from 1900/01/01 to 2015/08/31; Humans", 115530

#2,"Search chronic pain Filters: Publication date from 1900/01/01 to 2015/08/31; Humans", 66528

#1,"Search randomized controlled trial Filters: Publication date from 1900/01/01 to 2015/08/31; Humans", 489634
