# Supplementary material for: What is the effect of sensory discrimination training on chronic low back pain? A systematic review
Source: BMC Musculoskelet Disord. 2016 Apr 2;17:143. doi: 10.1186/s12891-016-0997-8 (PMC4818915; doi:10.1186/s12891-016-0997-8)
Supplement: Additional file 3: — Summary of included studies. (DOCX 28 kb) [file 12891_2016_997_MOESM3_ESM.docx]

**Additional File 3: Summary of included studies**

| Study | Methods | Participants | Interventions | Outcome Measures | Notes |
| --- | --- | --- | --- | --- | --- |
| Barker et al 2008 [15] | - Single-blinded, randomised controlled, non-inferiority trial  - Setting: Physiotherapy Department at the Nuffield Orthopaedic Centre NHS, Oxford, United Kingdom  - Informed consent: yes  - Ethics approval: yes | 60 patients, referred to a Physiotherapy Department with diagnosed CLBP  Inclusion criteria:  - >18 years  - diagnosed CLPB lasting for a minimum of 3 months  Exclusion criteria:  - patients with leg pain  - current TENS users  - patients which might be harmed by either of the treatment modalities (This was based on the warnings and precautions for the use of TENS, namely patients with a pacemaker, damaged or broken skin, malignancy, poorly enervated areas and spinal infection.)  It was not mentioned if all inclusion criteria were met (e.g. surgery) | 1) FairMed (intervention group)  The FairMed device: This device consists of two components: a hand held controller with subject interface and an array including 16 vibrating stimulation points which can be placed on the lumbar spine. These vibrating points can be activated separately. The user interacts with the controller indicating which point has been stimulated. The device than responds whether the answer is correct or incorrect (it is not mentioned whether the point is stimulated again if the answer is incorrect). Patients have been instructed to use the device for 30-minutes-sessions.  2) TENS (control group)  For the TENS group, a portable TENS TPN 200 PLUS unit has been used, applying conventional TENS with parameters of 80 Hz and 100 µs. Patients were suggested to use the TENS as much and as often as required. Two surface electrodes were placed (5cm × 5cm) at a distance of 5cm – 20cm to the painful area. The intensity has been adjusted in order to produce a tingling sensation, which would be approximately 2 – 3 times the sensory threshold.  Duration:  Intervention group: sessions of 30 minutes (not mentioned how often) / 3 weeks  Control group: as often as required / 3 weeks | 1) Pain: VAS (0 – 100mm)  2) Function: Oswestry disability index (ODI), (0-100)  Levels of pain and function were assessed pre and post treatment  Analysis:  Levels of pain and function have been assessed at baseline and after 3 weeks, 6 weeks and 12 weeks (results after 6 and 12 weeks are not noted, neither mentioned in the result part). | Significant problems with the durability of the FairMed, with 20/32 patients reporting a fault at some point in the intervention phase  - Funding: not reported |
| Hohmann et al. 2012  [20] | - Randomised controlled pilot study  - Setting: Department of Complementary and Integrative Medicine, Kliniken Essen-Mitte, Essen, Germany  - Informed consent: yes  - Ethics approval: not mentioned | 42 patients were recruited using flyers and announcement on the institutional homepage.  Inclusion criteria:  - 18 – 75 years  - CLBP for at least three consecutive months  Exclusion criteria:  - radicular pain  - neurological symptoms suggesting a disc prolapse  - vertebral column surgery less than 12 months prior to the study  - chiropractic manoeuvre or infiltration at the area treated four weeks prior to the inclusion in the study  - congenital deformation of the spine  - insulin-dependent diabetes mellitus  - dermatological diseases or skin changes at the treated area  - Severe mental illness that required medication  - a known tendency for haemorrhages  - Current anticoagulation or corticosteroid medication.  It was not mentioned if all inclusion criteria were met (e.g. pregnancy) | 1) Intervention group:  The intervention consisted of a home training with a mechanical needle stimulation pad. This is a plastic mat consisting of 60 hexagonal discs, with 19 spikes each.  The participants were instructed to use this device daily at home during 14 days.  Procedure: The patients had to press both feet on the above-described mat for 10 minutes while sitting on a chair (no detailed procedure). Afterwards it was recommended to put the needle stimulation pad on a soft surface, e.g. the bed, and lie on the top of it with the uncovered painful part of the back. The participants were informed that the first 2 to 5 minutes could be painful and it was recommended to treat the painful area of the back for about 30 minutes (no detailed procedure).  2) Waiting list control group:  Received no therapy (no details available)  They received the intervention too after having participated to this study (results not documented).  Duration:  Daily treatment of 30 minutes was recommended / 14 days | 1) Pain: NRS (0-10)  2) Function: ODI (0-100)  3) Mechanical detection threshold (not included in this SR)  4) Pressure pain threshold (not included in this SR)  5) Vibration detection threshold (not included in this SR)  Analysis:  The assessments were taken after the intervention had finished (14 days after the baseline assessment). | - Funding: supported by the Karl and Veronica Carstens Foundation  Product information: Zhencidian pad, CMP Chinese Medical Products Trading GmbH, Austria |
| Morone et al. 2011 [16] | - Randomised clinical study  - Setting: outpatient academic hospital in Rome, Italy  - Informed consent: yes  - Ethics approval: yes | 75 patients of an academic hospital  Inclusion criteria:  - 18 – 75 years  - CLBP persisting for at least three months  Exclusion criteria:  - Acute LBP  - pain due to a specific cause (e.g. fracture, spondylolisthesis, disc herniation and lumbar stenosis), scheduled back surgery, severe cognitive impairments, pregnancy, presence of concomitant rheumatologically, neurological, psychiatric, cardiological, respiratory, oncological diseases that could affect spine function or alter the perception of pain.  It was not mentioned if all inclusion criteria were met (for example surgery) | 1) Surface perceptive group (intervention group)  The “Surface for Perceptive Rehabilitation” was developed as a therapeutic system with small latex cones which are fixed on a support surface. These cones, normally 100 are used; vary in dimension (height 3-8 cm, base diameter 2-4cm) and elasticity (capacity to change in volume from 20% up to 60%). They have been put in standardized order according to previous studies (unspecified) for the first session. The participants were lying supine on this cone surface which stimulated the body. After the first session the hyperaemia areas were documented and then the cone surface for the next session was adapted in order to get an improved symmetry between back and surface (not explained in detail). In the following sessions the patient underwent cognitive-perceptive rehabilitation to interact actively with the cone surface (training explained but not in details). Normally, a session lasted 45 minutes and began with relaxation (not written how long), and then the patient performed active exercises, consisting of tactile and proprioceptive tasks with increasing difficulty. The exercises are listed in the study, but it is not mentioned in which order and dose they have been applied and according to which criteria the difficulty was increasing.  2) Back school program group (control group 1)  The Back school program group has been treated by an intensive intervention comprising theory lessons including anatomical, psychological and ergonomically knowledge. Pamphlets covering these topics as well as information for daily life, for example suggesting the correct posture at work, have been handed out. Nine sessions focused on exercises of breathing, stretching of trunk muscles, erector spine and abdominal reinforcement and postural exercises. The treatment is referenced to be based on [40] but not explained in detail.  3) group with medical and pharmacological assistance only (control group 2)  The participants of the control group 2 received only medical and pharmacological assistance (as the other two groups) such as analgesics, miorelaxants and nonsteroidal anti-inflammatory drugs.  Duration:  Intervention group: 3 session (45 minutes) per week during 1 month  Control group 1: 3 sessions per week during 4 weeks (approximately the same treatment duration as for the intervention group)  Control group 2: no treatment | 1) VAS (0-100mm)  2) MPQ (0-100)  3) ODI (0-100)  4) WDI (0-9)  All measurements were recorded before treatment, at the end of the treatment, 12 and 24 weeks after.  Analysis:  Levels of pain and function have been assessed at baseline, post-treatment, after 12 weeks and after 24 weeks. | - Funding: received no specific grant from any funding scheme |
| Paolucci et al 2012  [37] | - RCT  - Setting: academic hospital in Rome, Italy  - Informed consent: yes  - Ethics approval: yes | 30 patients of an academic hospital Recruitment: not reported  Inclusion criteria:  - 18-75 years  - diagnosed CLBP (back pain without any specific cause that lasts longer than 12 weeks)  Exclusion criteria:  - Acute LBP  - pain due to a specific cause (e.g. fracture, spondylolisthesis, disc herniation and lumbar stenosis), scheduled back surgery, severe cognitive impairments, pregnancy; presence of concomitant rheumatological, neurological, psychiatric, cardiological, respiratory or oncological diseases that could affect spine function or alter the perception of pain.  It was not mentioned if all inclusion criteria were met (e.g. surgery, inclusion/exclusion for healthy control group) | 1) Su-Per treatment  Same [16]  2) Back school group  Same as control group in [16]  3) Healthy individuals as control group received no treatment  No intervention (not included in the SR)  Duration:  Intervention group: 3 sessions (45 minutes) per week during 1 month  Control group: 3 sessions (45 minutes) per week during 1 month (approximately the same treatment duration as for the intervention group) | 1) Stabilometric assessment (not integrated in this SR)  2) McGill Pain Questionnaire (range not mentioned. Pain rating index: 0 – 100)  Analysis:  Levels of pain have been assessed at baseline and at the end of the treatment. | - Funding: not reported |
| Ryan et al 2014 [38] | - pilot randomised controlled trial  - Setting: NHS physiotherapy outpatient department within a UK hospital  -Informed consent: yes  -ethic approval: yes | 24 patients  Study invitations were sent to all potentially suitable individuals with CLBP (via mail)  Inclusion criteria:  - ≥ 18 years  - pain duration ≥ 6 months  - CLBP ≥6 months with/without leg pain  Exclusion criteria:  - not having an informal carer to assist the home training program  - being unable to read English: therefore isolated persons and those not understanding English are not covered in this study  It was not mentioned if all inclusion criteria were met (e.g. surgery, pregnancy) | The intervention- and the control group received three sessions (stimulation or acuity training) with a physiotherapist and followed their home training program with an informal carer. In addition, both groups had usual care physiotherapy (not reported in detail).  1) Tactile acuity training intervention (intervention group)  The Tactile acuity training intervention comprised two components:  3 blocks of 24 stimuli have been performed over approximately 24 minutes.  a) Tactile acuity training:  This training consisted of marking five or ten sites of the painful area which then have been randomly stimulated by a big or a small probe. The participant had to concentrate on the stimulus and tell which of the points was stimulated and which probe was used. If > 90% of the answers were correct, the marks were moved 10% closer to make the task more difficult. The same program was performed at home as part of the home training program.  b) Graphaesthesia acuity training:  Graphaesthesia acuity training consisted of a series of 60 letters of the alphabet which have been traced on the painful area by the clinician or the carer. They were about 1 inch high. The patient had to identify the letter and was given guided feedback. He or she was informed whether his/her answer was correct and if not, the letter was retraced and the patient was informed about the correct answer.  2) Tactile stimulation (sham group)  The placebo group (sham) received the same tactile stimulation as the intervention group, but they did not focus on the stimulus and therefore had no interaction with the carer.  For the first treatment of the patient, the carer was invited to participate as well, in order to be taught about his role.  The aim was that every patient received a total of 21 sessions of therapy, of which 3 sessions (intervention or placebo) were provided by the physiotherapist.  Follow-up: is recorded. The intervention group lost 3 participants; Placebo lost 6 participants, which is quite a lot out of 24.  Duration:  Minimal duration: 21 days (21 sessions)  Maximal duration: decided by the clinician providing the usual care | 1) Pain: VAS (0 – 100mm)  2) Function: RMDQ, (0 – 24)  Levels of pain and function were assessed pre- and post-treatment  Analysis:  Levels of pain and function have been assessed at baseline and post-treatment. | - Funding: not reported  In the tactile stimulation (sham) group, 6 patients were lost (for several reasons), which is half of this group’s test persons, considering the group size of n=12. |
| Vetrano et al. 2013 [39] | - Single-blind, RCT  - Setting: outpatient academic hospital in Rome, Italy  - Informed consent: yes  - Ethics approval: yes | 24 patients with CLBP  The recruited patients had consulted one the participating physicians from January to July 2011.  Inclusion criteria:  - 25-70 years  - Established diagnosis of CLPB not attributable to a recognizable, known specific pathology (e.g. infection, neoplasisa, osteoporosis, fracture, structural deformity, inflammatory disorder – e.g. ankylosing spondylitis – radicular syndrome or cauda equina syndrome) for at least 12 weeks before the treatment  - capable of completing questionnaires and of giving informed consent  - a “wash-out” period of 12 weeks was required between any non-operative therapy and the inclusion in the study  Exclusion criteria:  - History of surgery on the spine and/or abdominal surgery, abdominal aortic aneurysm, presence of herniated lumbar disc, spondylosthesis, spinal stenosis, serious and severe scoliosis or kyphosis, previous osteoporotic fractures based, cancers, systematic rheumatic, cardiological, respiratory and neurological diseases, pregnancy and diagnosis of psychiatric disorders | 1) Standard Su-Per treatment (intervention group 1)  Same as [16] except for the duration which is 5min less per session.  2) Su-Per treatment without higher stimulus at the interspinous line (Intervention group 2 / control group)  Same as Group 1, but also more deformable cones in the midline.  Duration:  Intervention group: 3 session (40 minutes) per week during 1 month  Control group: received the same dose of therapy as the intervention group. | 1) Pain: VAS (0 – 100mm)  2) McGill Pain Questionnaire  3) ODI (0-100)  All measurements were recorded before treatment, at the end of the treatment, 4 and 12 weeks after.  Analysis:  Levels of pain and function have been assessed at baseline, post-treatment, after 4 weeks and after 12 weeks. | - Funding: not reported |

Abbreviations:

CLBP: chronic low back pain; LBP: Low Back Pain; MPQ: McGill Pain Questionnaire; NHS: National Health Service; NRS: Numeric Rating Scale; ODI: Oswestry Disability Index; RCT: randomized controlled trial; RMDQ: Roland Morris Disability Questionnaire; SR: systematic review; SuPer: Standard surface for perceptive rehabilitation; TENS: transcutaneous electrical nerve stimulation; VAS: Visual Analog Scale; WDI: Waddlle Disability Index
